# Executive and memory dysfunction related to binge drinking in stroke survivors during a 9-year follow-up

**DOI:** 10.3389/fnagi.2024.1360236

**Published:** 2024-03-15

**Authors:** Siiri Laari, Tatu Kauranen, Katri Turunen, Satu Mustanoja, Turgut Tatlisumak, Erja Poutiainen

**Affiliations:** ^1^Department of Psychology and Logopedics, Faculty of Medicine, University of Helsinki, Helsinki, Finland; ^2^HUH Neurocenter, Helsinki University Hospital and University of Helsinki, Helsinki, Finland; ^3^Department of Neurology and Clinical Neurophysiology, Lapland Central Hospital, Rovaniemi, Finland; ^4^Institute of Neuroscience and Physiology, Sahlgrenska Academy at the University of Gothenburg and Department of Neurology, Sahlgrenska University Hospital, Gothenburg, Sweden

**Keywords:** executive dysfunction, memory, ischemic stroke, binge drinking, long-term cognitive outcome, cognitive impairment, follow-up, stroke survivor

## Abstract

**Background:**

Ischemic stroke and heavy alcohol consumption are both known risk factors for cognitive impairment. The issue gains importance because the prevalence of stroke and binge drinking have both increased among working-aged adults. Alarmingly, a recent cross-sectional study suggests the additive negative effects of binge drinking and comorbid brain disease on cognition. However, the long-term cognitive prognosis of the additive effects of stroke and binge drinking on adults remains unknown.

**Methods:**

In this prospective, two-center cohort study, we recruited consecutive 18–65-year-old patients with first-ever ischemic stroke along with demographically matched stroke-free controls. Patients participated in neuropsychological assessment at 6 months, 2 years, and 9 years after stroke, and in neurological assessment at acute care and at 9-year follow-up. Controls participated in a similar follow-up procedure. We examined the association between binge drinking, follow-up time, and long-term cognitive outcomes using repeated-measures analysis of variance.

**Results:**

We included 85 patients who had had their first-ever and only ischemic stroke (mean age 53 years at the incident stroke). Patients were divided into binge-drinking (*n* = 22) and non-binge-drinking groups (*n* = 63) based on the shortened version of the Alcohol Use Disorders Identification Test. Follow-up data in healthy controls (*n* = 31) was used to normalize the patients' test scores for effects of age, sex, and education. We compared cognitive changes between binge-drinking and non-binge-drinking patients over a 9-year follow-up. Non-binge-drinking patients outperformed binge-drinking patients across all follow-up points on most of the executive function tests and in one memory test: binge drinking had a significant main effect both on executive function (the phonemic fluency task, *p* = 0.002; the Trail Making Test, *p* = 0.013) and memory (the list learning task, *p* = 0.002).

**Conclusion:**

Binge drinking was associated with executive and memory dysfunction at three time points over a decade after a first-ever ischemic stroke. Subdiagnostic binge drinking might increase the adverse effects of a first-ever ischemic stroke on executive function and memory, evident over a decade poststroke.

## 1 Introduction

Binge drinking leads to multisystemic pathophysiological consequences in the body, including structural changes in the brain (Molina and Nelson, 2018). Increased binge drinking has become a more prominent issue in public health among working-aged adults in the United States (Grucza et al., 2018). Binge drinking, or heavy episodic drinking pattern, refers to the consumption of five or more standard drinks (13.5 g of ethanol) within 2 h (National Institute on Alcohol Abuse and Alcoholism, 2004).

Subdiagnostic binge drinking without an alcohol use disorder (AUD) is proposed to represent a less severe end in a continuum of alcohol-related cognitive disorders (Stephens and Duka, 2008). What is known about the link between cognitive impairment and subdiagnostic binge drinking is largely based on studies in adolescents. There is growing evidence of the cognitive consequences of binge drinking in the developing brain (Mota et al., 2013; Winward et al., 2014a; Morris et al., 2018). Research on adolescents has shown that deficits in executive functions (regulating goal-directed actions) are associated with a history of frequent binge drinking (Squeglia et al., 2012; Winward et al., 2014a,b). Imaging studies on adolescents indicate binge drinking-related structural changes in the prefrontal and parietal regions, accompanied by executive dysfunction (Morris et al., 2018). Binge-drinking adolescents have also demonstrated verbal memory dysfunction partially attributed to their executive dysfunction (Parada et al., 2011; Mota et al., 2013; Winward et al., 2014b). Furthermore, it has been suggested that heavy alcohol use is associated with poor learning and verbal memory over time in a 10-year longitudinal study that followed youth from the age of 16 (Hanson et al., 2011a,b).

Regarding working-aged adults, very few studies have suggested a link between cognitive deficits and subdiagnostic binge drinking. First, adult truck drivers who reported a history of binge drinking performed poorer in executive function tasks than non-binge-drinking peers (de Oliveira et al., 2016). Second, the rest of the studies recruited participants with comorbid brain diseases. For example, a recent cross-sectional study found an additive negative effect of binge drinking and human immunodeficiency virus (HIV) on, e.g., learning and memory skills (Paolillo et al., 2022). Binge drinking and ischemic stroke had an additive negative effect on executive function during the subacute phase of stroke in our previous study (Laari et al., 2020). Cognitive impairment is a common consequence of stroke. These cross-sectional studies suggest that in adults, cognitive deficits related to binge drinking might be subtle and become evident after another brain injury, such as an ischemic stroke. However, the long-term cognitive prognosis of binge drinking and its effects on recovering from comorbid brain injury in adults remains unknown.

Our hypothesis is that subdiagnostic binge drinking is associated with long-term deficits in executive function and verbal memory in patients with a history of the first-ever and only stroke. Second, we hypothesized that binge drinking is linked to executive function and memory decline over the 9-year follow-up. We compared executive function and memory between binge-drinking and non-binge-drinking patients at 6 months, 2 years, and 9 years poststroke, along with healthy demographic controls.

## 2 Methods

### 2.1 Stroke cohort and follow-up study design

Patients were part of a consecutive stroke inpatient cohort recruited from Helsinki University Central Hospital and Lapland Central Hospital from April 2007 to October 2009. The inclusion criteria were a first-ever diagnosis of supratentorial ischemic stroke, age 18–65 years, no severely altered state of consciousness or relevant history or comorbidity of neurological or psychiatric disease, and Finnish as a native language. The healthy controls consisted of spouses or relatives of the patients who were similar in terms of demographics to the patients. Controls met all the criteria that were set for the patients, except for having an ischemic stroke.

For this study, we excluded patients with recurrent stroke, other neurological diseases, and alcohol or substance use disorder at any point along the 9-year follow-up time. For a more comprehensive clarification of patients who were excluded or dropped out, refer to the flowchart in Laari et al. (2022). Furthermore, we included only those participants who completed all three follow-up visits. Patients were prospectively followed up at 6 months, 2 years, and 9 years after the index event. Controls were followed up first at a 3-month interval and then at a 9-year interval from the first visit. The Ethics Committee of Helsinki University Central Hospital approved the study and consent procedure (approval number 356/2017), and all participants signed an informed consent form.

### 2.2 Binge drinking

The shortened version of the Alcohol Use Disorders Identification Test (AUDIT) served as the foundation for our diagnostic criteria for binge drinking (Saunders et al., 1993). Shortened AUDIT includes average alcohol drinking frequency and an average number of standard drinks (1.65 standard units) containing 13.5 g of ethanol per typical drinking occasion over the last year (Aalto et al., 2006). The frequency of the drinking occasions was not specified. Shortened AUDIT was performed both at the acute care hospital (both by a stroke neurologist and by a neuropsychologist as a part of the research protocol) and at the 9-year follow-up study for patients. Healthy controls were assessed similarly and at respective time points.

The binge-drinking group consisted of patients who reported consuming five or more alcoholic drinks per occasion on a typical basis within the year prior to the incident stroke and/or within the year prior to the 9-year follow-up study. Similarly, the non-binge-drinking group consisted of patients who reported consuming four or fewer (0–4) drinks per occasion on a typical basis both within the year prior to stroke and the year prior to the 9-year study. Additionally, participants were required to abstain from alcohol for at least 24 h before each neuropsychological assessment.

### 2.3 Ischemic stroke data

At the acute care hospital, we collected neurological data to confirm the stroke diagnosis, along with potential confounders and effect modifiers. An experienced stroke neurologist specified ischemic supratentorial stroke diagnoses with brain imaging and neurological measures. All patients underwent brain imaging within the first few days after stroke with magnetic resonance imaging (MRI) and/or brain computed tomography (CT). The neurologist evaluated lesion locations, the lesion size (in millimeters from the plane in which the largest diameter was observed), and age-related white matter changes according to a widely used scale (Wahlund et al., 2001). The neurologist used the Barthel Index (Mahoney and Barthel, 1965) to evaluate physical functional status, the National Institutes of Health Stroke Scale (NIHSS) (Goldstein et al., 1989) to measure the severity of stroke at the time of discharge from the acute care hospital, and the Trial of Org 10172 in Acute Stroke Treatment (TOAST) criteria (Adams et al., 1997) to determine the stroke etiology (large-artery atherosclerosis, cardioembolic, small-artery occlusion, other determined, or undetermined). Stroke risk factors (including cigarette smoking, diabetes mellitus, serum cholesterol, coronary artery disease, atrial fibrillation, hypertension, myocardial infarction, and cardiac failure) were collected from the patient's case history and included in the structured interviews during acute care. In addition, the structured interviews covered age, education status, history of attention and learning disabilities, history of mood symptoms, history of neurologic and psychiatric diseases, AUD or substance use disorders, current use or history of medications, previous transient ischemic attacks, the Barthel Index, NIHSS, and structured interviews for stroke risk factors were repeated at the 9-year follow-up.

### 2.4 Neuropsychological data

To compare cognitive outcomes between binge-drinking and non-binge-drinking patients over a 9-year follow-up period, we used three executive functions and three memory tests. An experienced neuropsychologist conducted an assessment according to a strict protocol at each of the three follow-up visits. *Executive function* comprised the phonemic fluency task (Lezak et al., 2012); the Trail Making Test (TMT), the difference time score of parts B and A (Reitan, 1958); and the Stroop test, the difference time score of the interference and naming parts (Golden, 1978). *Memory* comprised the Logical Memory Tests I and II (immediate and delayed recall of a story) subtests of the Wechsler Memory Scale–Revised (WMS-R) (Wechsler, 1987) and the 10-word list-learning task (Lezak et al., 2012). In addition, we assessed mood states (e.g., symptoms of anxiety and depression) with the modified Profile of Mood States (POMS) questionnaire at every follow-up visit (McNair and Lorr, 1964).

We calculated patients' Z-scores for each test based on the controls' test score statistics. For normalizing the patients' baseline examination scores, we used the controls' first examination. For normalizing patients' 6-month and 2-year examination scores, we used the controls' second examination to control for learning effects. When controls' test scores were not strictly normally distributed, we checked that the means and standard deviations (SDs) were in accordance with available Finnish normative data (e.g., Wechsler, 1996).

We calculated patients' Z-scores for each test based on the controls' test score statistics. For normalizing the patients' 6-month assessment scores, we used the controls' first assessment. Similarly, for normalizing patients' 2-year assessment scores, we used the controls' second examination to control for learning effects. For normalizing patients' 9-year assessment scores, we used the controls' third examination performed 9 years from the first study to control for normal aging-related effects in cognitive function.

### 2.5 Statistical methods

We used SPSS version 28 (IBM, Armonk, NY, United States) software for statistical analyses. When necessary, we used basic transformations to obtain variable normality. The missing values were not replaced. The demographic and clinical variables were compared using an independent *t*-test and a chi-square (χ^2^) test between binge-drinking and non-binge-drinking patients.

In our main analyses, our statistical design involved a follow-up study conducted at three specific time points (6 months, 2 years, and 9 years poststroke). We compared the change in binge-drinking stroke patients' normalized cognitive test performance Z-scores to the change in non-binge-drinking patients' Z-scores during the three follow-up assessments. First, we performed a two-way repeated measures analysis of variance (ANOVA) for each cognitive test, with follow-up time as the repeated within-subject factor and binge drinking (binge vs. non-binge) as the predictor variable. Second, we incorporated those potential confounders that differed between drinking groups. We used Greenhouse-Geisser correction in cases of sphericity violations. When ANOVA indicated the main effect of binge drinking, we compared differences between drinking groups at each follow-up time point with an independent-sample *t*-test. We calculated G power to estimate the minimum number of subjects for adequate study power. A power analysis for the *F*-test indicated that the minimum total sample size to yield a statistical power of at least 0.8 with an alpha of 0.05 and a medium effect size (*d* = 0.5) is 52 (3:1). The statistical significance level was set at *p* < 0.05.

## 3 Results

### 3.1 Demographics and ischemic stroke characteristics

Overall, 85 stroke patients and 31 healthy controls were included in the follow-up study. The mean follow-up duration was 9 years for both patients and controls.

A comparison of demographics and ischemic stroke characteristics between binge-drinking and non-binge-drinking patients included in this study is presented in Table 1. Binge-drinking stroke patients were more often men (91% vs. 52%), had fewer years of education, consumed more alcohol doses per week (by definition), and had a lower mood state at 9-year follow-up as measured with POMS. To adjust the main analyses for potential confounders that differed between groups, we normalized the patients' test scores for effects of age, sex, and education using healthy controls follow-up data from respective follow-up time points. In addition, to control for the main effects of binge drinking for confounding factors, i.e., mood states that differed between drinking groups, we added the Profile of Mood States scores at 9-year follow-up as a covariate to the analysis.

**Table 1:** A comparison of demographics and clinical characteristics between binge-drinking and non-binge-drinking stroke patients at the time of the incident stroke and at the 9-year follow-up.

	**Binge (*n* = 22)**	**Non-binge (*n* = 63)**	***P*-value**
**At the time of the incident stroke**			
Sex, men^‡^	20 (90.9)	32 (51.6)	0.001
Age, years (SD)^†^	53.6 (8.2)	53.1 (11.5)	0.420
Education, years (SD)^†^	11.5 (2.2)	12.9 (2.8)	0.014
History of attention disorder^‡^	3 (13.6)	2 (3.2)	0.073
History of learning disorder^‡^	4 (18.2)	13 (20.6)	0.804
Premorbid mood symptoms^‡^	3 (13.6)	6 (9.5)	0.589
Mood state, POMS score^†^	47.2 (22.1)	40.7 (22.4)	0.005
NIHSS score^†^ (Goldstein et al., 1989)	0.59 (0.85)	0.29 (1.0)	0.495
Barthel Index score^†^ (Mahoney and Barthel, 1965)	99.5 (1.6)	96.3 (15.8)	0.189
**Infarct characteristics**			
White matter changes^‡^	11 (50.0)	21 (34.0)	0.181
Silent infarctions^‡^	7 (31.8)	13 (21.0)	0.305
Infarct size, largest diameter in mm^†^	23.9 (21.5)	22.5 (25.6)	0.409
Infarct side left/right^‡^	14/6 (70/30)	21/21 (50/50)	0.138
**Infarct location**			
Cortical^‡^	8 (36.4)	18 (29)	0.523
Subcortical^‡^	9 (40.9)	16 (25.8)	0.183
Frontal^‡^	2 (9.1)	10 (16.1)	0.418
Parietal^‡^	5 (22.7)	20 (32.3)	0.401
Temporal^‡^	8 (36.4)	15 (24.2)	0.271
Occipital^‡^	5 (22.7)	12 (19.4)	0.735
Basal ganglia^‡^	6 (27.3)	13 (21.0)	0.544
Several locations^‡^	8 (36.4)	13 (20.6)	0.141
**Stroke etiology by TOAST criteria (Adams et al.**, **1997****)**			0.407
Large-artery atherosclerosis^‡^	6 (27.3)	11 (17.7)	
Cardioembolism^‡^	3 (13.6)	15 (24.2)	
Small-artery occlusion^‡^	2 (9.1)	14 (22.6)	
Other determined etiologies^‡^	4 (18.2)	8 (12.9)	
Undetermined etiologies^‡^	7 (31.8)	14 (22.6)	
**Stroke risk factors**			
Diabetes mellitus^‡^	5 (22.7)	14 (22.2)	0.961
High serum cholesterol^‡^	14 (63.6)	39 (61.9)	0.885
High blood pressure^‡^	12 (54.5)	40 (63.5)	0.459
Atrial fibrillation^‡^	5 (22.7)	13 (20.6)	0.836
Coronary artery disease^‡^	1 (4.5)	12 (19.0)	0.104
Myocardial infarction^‡^	1 (4.5)	5 (7.9)	0.593
Cardiac failure^‡^	2 (9.1)	4 (6.3)	0.666
Cigarette smoking^‡^	6 (27.3)	10 (15.9)	0.239
Alcohol doses weekly^†^	11.9 (11.1)	3.3 (4.4)	0.001
Other substance use^‡^	1 (4.5)	1 (1.6)	0.431
**At the 9-year follow-up**			
Mood state, POMS score^†^	14.5 (3.2)	20.3 (2.7)	0.005
ADL difficulties^‡^	3 (14.3)	12 (20.0)	0.562
Barthel Index score^†^	98.9 (8.0)	98.1 (2.6)	0.325
NIHSS score^†^	0.59 (0.85)	0.29 (1.0)	0.495
TIA during follow-up^‡^	3 (13.6)	5 (8.1)	0.444
**Stroke risk factors**			
Diabetes mellitus^‡^	5 (22.7)	13 (21.0)	0.863
High serum cholesterol^‡^	14 (63.6)	39 (62.9)	0.951
High blood pressure^‡^	12 (54.5)	40 (64.5)	0.408
Atrial fibrillation^‡^	5 (22.7)	13 (21.0)	0.863
Coronary artery disease^‡^	1 (4.5)	12 (19.4)	0.099
Myocardial infarction^‡^	1 (4.5)	5 (8.1)	0.582
Cardiac failure^‡^	2 (9.1)	4 (6.5)	0.680
Cigarette smoking^‡^	6 (27.3)	10 (16.1)	0.253
Alcohol doses weekly^†^	7.1 (9.4)	2.3 (3.2)	< 0.001
Other substance use^‡^	1 (4.5)	1 (1.6)	0.438

^†^Compared with the t-test, mean (SD), ^‡^Compared with the chi-square (χ^2^) test, n (%).

NIHSS, National Institutes of Health Stroke Scale at discharge; TOAST, the Trial of Org 10172 in Acute Stroke Treatment.

### 3.2 Executive function

**Figure 1:**
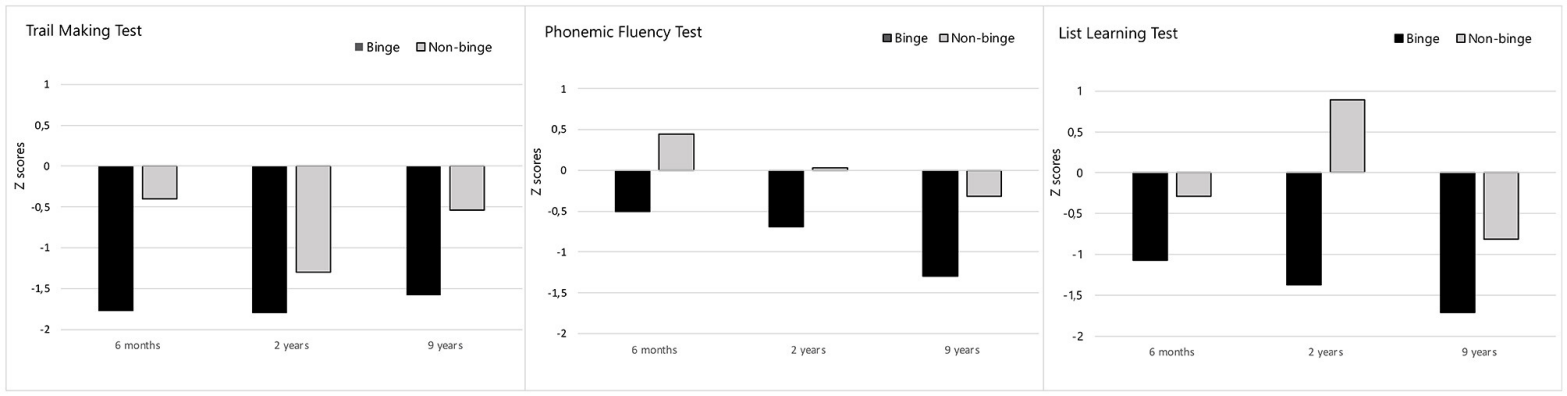
The Phonemic fluency, the Trail Making, and the List Learning Z-scores at different follow-up time points. Binge-drinking patients' performance (black) compared to non-binge-drinking patients' performance (light gray). The Y-axis represents three follow-up time points (6 months, 2 years, and 9 years poststroke). The X-axis represents patients' Z-scores based on the controls' test score statistics. A Z-score of 0 indicates that the data point's score is identical to the healthy controls' mean score, and a Z-score of −1.0 indicates a value that is below one standard deviation from the mean.

In executive function, the main effect of binge drinking was significant for the phonemic fluency task and the Trail Making Test but not for the Stroop test (Supplementary Table 1). Non-binge-drinking patients outperformed binge-drinking patients on the phonemic fluency task (*p* = 0.002) and the Trail Making Test (*p* = 0.013) (Figure 1). This means that executive function performance was significantly weaker in binge-drinking patients across all follow-up points. Adding the POMS score as a covariate to adjust the analysis for mood state did not change the main effects of binge drinking on executive function. There was neither a significant main effect nor an interaction effect of mood state on executive function.

To confirm the main effects of binge drinking separately at each follow-up time point, we did *post hoc* comparisons between drinking groups separately at every time point. As for the phonemic fluency task, non-binge-drinking patients outperformed binge-drinking patients at all time points (6 months, 2 years, and 9 years poststroke) [t_(83)_ = 2.67, *p* = 0.005; t_(83)_ = 2.92, *p* = 0.002; t_(83)_ = 3.56, *p* < 0.001, respectively]. As for the Trail Making Test, *post hoc* comparisons confirmed that difference time was longer in binge-drinking patients compared to non-binge-drinking patients both at 6 months and 9 years poststroke [t_(83)_ = −3.17, *p* = 0.001; t_(83)_ = −2.50, *p* = 0.007, respectively].

However, the interaction effects between binge drinking and follow-up time for executive tests were nonsignificant. This means that the cognitive decline rate over the course of a 9-year follow-up did not differ between binge-drinking and non-binge-drinking groups.

Finally, the main effects of follow-up time on executive function tests are presented in Supplementary Table 1. The main effects of time indicated that the performance of both binge and non-binge groups declined over time on the phonemic fluency task, the Trail Making Test, and the Stroop test. As seen in Figure 1, especially the performance in phonemic fluency declined during the 9-year follow-up in all stroke patients, with a Z-score of 0 representing the average performance of healthy controls.

### 3.3 Memory

The main effect of binge drinking was significant for the list-learning task but not for Logical Memory Tests I and II (Supplementary Table 1). Non-binge-drinking patients outperformed binge-drinking patients on the list-learning task (*p* = 0.002) across all follow-up points. Adding the POMS score to control for mood state did not change the main effects of binge drinking on list learning. There was neither a significant main effect nor an interaction effect of mood state on memory.

*Post hoc* comparisons confirmed the interpretation of the main effect of binge drinking on list learning as binge drinking-related differences at each time point: non-binge-drinking patients outperformed binge-drinking patients at all time points (6 months, 2 years, and 9 years poststroke) on the list-learning task [t_(82)_ = 2.62, *p* = 0.005; t_(82)_ = 3.69, *p* < 0.001; t_(82)_ = 2.32, *p* = 0.011, respectively].

Regarding the main effect of follow-up time, the performance of both groups declined over time on the Logical Memory Tests I and II and the list-learning task (Supplementary Table 1). As seen in Figure 1, there was a noticeable decline in the list learning performance among all stroke patients during the 9-year follow-up.

Moreover, there was an interaction effect between binge drinking and follow-up time [F_(2, 164)_ = 3.40, *p* = 0.036, $\eta_{\text{p}}^{2}$ = 0.040] such that binge-drinking patients' performance had a descending trend toward a 9-year study, but non-binge-drinking patients performed better at a 2-year follow-up than a 6-month or 9-year follow-up (Figure 1). However, the following subsequent analyses of differences between groups were non-significant.

## 4 Discussion

This study set out to investigate the additive effects of stroke and binge drinking on long-term cognitive prognosis. In line with our hypothesis, the binge-drinking group showed more pronounced executive and verbal memory dysfunction than the non-binge-drinking group at every time point over the 9-year follow-up. In addition, there was a trend toward memory decline related to binge drinking over the 9-year follow-up period.

Overall, executive function and memory performance declined over the 9-year follow-up period in all stroke patients, as could be expected. During the 9-year follow-up, both binge-drinking and non-binge-drinking patients experienced a decline in executive function and memory, which aligns with the effects of normal aging (Salthouse, 2010). Nevertheless, in the phonemic fluency test and the list learning task, the pattern of decline over a 9-year follow-up appeared to be more pronounced than could be expected due to normal aging.

The main finding in our study was that executive dysfunction related to binge drinking was evident at 6 months, 2 years, and 9 years after ischemic stroke (de Oliveira et al., 2016). Binge-drinking patients performed poorer than non-binge-drinking patients in the phonemic fluency test and the Trail Making Test. Binge-drinking patients came up with fewer words on the phonemic fluency test, and in the Trail Making Test, their difference time score was greater than that of the non-binge-drinking patients. Both tests measure one's capability for set-shifting (Miyake et al., 2000). In our previous study, we found a similar negative effect of binge drinking on phonemic fluency at the subacute phase, 3 months poststroke (Laari et al., 2020). Poor performance in phonemic fluency related to binge drinking has not previously been described in other studies. One previous study using the same task did not find differences between adolescent binge-drinking and non-binge-drinking groups (Parada et al., 2012). Although the phonemic fluency task has been less used in previous studies, our results of both set-shifting tasks are broadly consistent with earlier findings in adolescents (Squeglia et al., 2012; Winward et al., 2014b,c). Regarding the other set-shifting task we used, our result supported the previous findings of poor performance in the Trail Making Test associated with the history of binge drinking in adolescents (Winward et al., 2014b,c; Salas-Gomez et al., 2016). We used the difference score to extract the effects of the visuomotor component of the task to get a better approximation of executive function shifting between sets. Overall, our results reflect impaired set-shifting ability and cognitive flexibility related to binge drinking. One possible explanation for this finding could be that both binge drinking and ischemic stroke damage partly similar brain networks that are distributed across the frontal areas underlying executive functions (Stuss, 2011). More specifically, set-shifting impairment is one of the executive dysfunctions traditionally associated with dorsolateral prefrontal cortex injury; however, it is now recognized that it can also result from the impaired parietal-temporal-frontal system (Jones and Graff-Radford, 2021). Thus, ischemic stroke in different areas could disturb set-shifting ability. In our study, patients in binge-drinking and non-binge-drinking groups did not differ according to the ischemic lesion characteristics, such as the site of the lesion. Thus, this interesting finding could be attributed to binge drinking. Based on our findings, chronic ischemic stroke can worsen executive dysfunction related to binge drinking, which might have been less noticeable before the stroke.

The second major finding was the association between binge drinking and verbal memory in the list-learning task. The number of unrelated words learned was consistently lower for binge-drinking than non-binge-drinking patients at 6-month, 2-year, and 9-year follow-ups. Our results mirror those of previous studies examining list learning by repetition in binge-drinking adolescents (Parada et al., 2011; Sanhueza et al., 2011). Even though previous studies in adolescents used longer word lists and were thus more challenging than lists in our study, impaired learning was still consistently linked to binge drinking in our study. Contrary to binge-drinking-related declines in list learning, we found no differences in recalling a story. A possible explanation for this might be that there is more implicit structure in a story than in an unrelated word list. Therefore, learning a list requires a more comprehensive use of strategies and executive functions than recalling a story. Our result is in line with the additive effects of binge drinking and comorbid brain disease on impaired learning (Paolillo et al., 2022). Furthermore, we found an interaction effect reflecting a more pronounced decline over time in binge drinking compared to the non-binge-drinking group in list learning. In further analyses, however, the cognitive decline rate over time did not significantly differ between binge-drinking and non-binge-drinking groups. Thus, with this data, we were unable to confirm or specify the timeline of decline related to binge drinking. The descending trend in learning over time related to binge drinking in this study is, however, consistent with previous studies in adolescents suggesting that heavy alcohol use might be associated with worsening verbal memory and learning over 10 years (Hanson et al., 2011a,b). In the future, a study with a larger sample size and a more demanding learning task could clarify the effects of subdiagnostic binge drinking on learning.

The binge-drinking group, on average, consumed 12 standard drinks of alcohol per week before the stroke and 7 drinks at the end of the follow-up. Thereby, the average alcohol consumption of the binge-drinking group was moderate and within the weekly limit for low-risk by a U.S. standard (National Institute on Alcohol Abuse and Alcoholism, 2004), although the binge-drinking group drank significantly more doses per week than the non-binge-drinking group. The standard acceptable alcohol intake is under debate and varies across countries. However, the standard acceptable weekly intake in Europe is approximately similar to that in the US (Wood et al., 2018). In our data, the moderate average alcohol intake in the binge-drinking group suggests that the cognitive deficits found in our study might relate to the binge pattern of alcohol drinking.

Our findings regarding the link between binge drinking and cognitive dysfunction in ischemic stroke patients are noteworthy due to the limited number of studies that have previously shown the cognitive consequences of binge drinking in adults (de Oliveira et al., 2016). In our stroke patient data, binge drinking was linked to impaired executive function and learning, despite the absence of an AUD. This is an interesting finding, as previous evidence of subdiagnostic binge drinking linked to cognitive impairment is mostly from adolescents. Regarding brain structure alterations associated with binge drinking without AUD, binge drinking in adolescents affects the developmental trajectories of brain structures, particularly in the frontal and temporal lobes (Lees et al., 2020). As for the effect of alcohol on the adult brain, previous studies have investigated the effects of AUD. AUD is a well-established cause of brain atrophy and dementia in adults. A long-term prospective study reported that a history of AUD doubled the odds of later severe memory impairment (Kuzma et al., 2014). Frontal white matter atrophy, white matter lesions, and hippocampal atrophy are consistent findings in individuals diagnosed with AUD (Topiwala et al., 2017). Compatible with brain areas affected, memory impairment is common among individuals with AUD, and over 80% have executive deficits (Giancola and Moss, 1998). The findings of this study suggest that engaging in binge drinking without an AUD could represent a less severe form of alcohol-related cognitive dysfunction along a continuum.

### 4.1 Strengths and limitations

By conducting a neuropsychological assessment at several time points, we were able to examine and understand how cognitive functions change over time. By including a healthy demographic control group with corresponding follow-up visits, we were able to account for the normal cognitive changes that occur with aging. In addition, for the normal effect of aging, we were able to adjust the observed cognitive changes for education and sex. To control the effects of ischemic stroke, we only included those first-ever stroke patients who did not experience a further stroke or comorbid brain disease (such as a tumor or dementia). In addition, none of the ischemic stroke-related variables, such as stroke size, location, previous white matter changes, or stroke risk, differed between groups.

These data must be interpreted with caution because of the small sample size, especially in the binge-drinking group. Moreover, there were more men in the binge-drinking group. We could not, however, add sex as a covariate in the analyses because the subgroups would have been too small for statistical analyses. Thus, the conclusions of our study are limited to men with ischemic stroke, as there were only two women in the binge drinking groups. Nevertheless, we normalized the patients' cognitive test scores using demographically (sex, age, and education) matched healthy controls' follow-up data from respective follow-up time points. An additional limitation of this study was that, due to the small sample size, we could not control for the possible effects of the history of attention disorder. A history of attention disorder may predispose to binge drinking because alcohol use and executive function are suggested to have a reciprocal link (Hicks et al., 2012). Although a higher percentage of binge-drinking patients than non-binge-drinking patients had a history of attention disorder in our study, the difference between groups was nonsignificant.

### 4.2 Conclusion

Our results suggest that binge drinking might increase the adverse effects of a chronic ischemic stroke on executive functions and verbal memory even for a decade after the first-ever stroke. Binge drinking was linked with impaired performance in neuropsychological tests measuring set shifting and learning at every time point along the follow-up. However, all stroke patients showed a cognitive decline during the 9-year follow-up period. This study on Finnish stroke patients emphasizes the cognitive risks associated with alcohol consumption that does not meet the criteria for AUD but instead falls within the range of moderate drinking. This is noteworthy, as executive function and memory impairment are known risk factors for returning to work after an ischemic stroke (Kauranen et al., 2013). These findings have important clinical and public health implications and suggest that it is worth considering the role of alcohol consumption in the clinical evaluation of conditions such as stroke.

## Data availability statement

Source data will not be made available because of legislative issues regarding patient privacy. Requests to access the datasets should be directed to siiri.laari@hus.fi.

## Ethics statement

The studies involving humans were approved by HUS Regional Committee on Medical Research Ethics. The studies were conducted in accordance with the local legislation and institutional requirements. The participants provided their written informed consent to participate in this study.

## Author contributions

SL: Conceptualization, Data curation, Formal analysis, Investigation, Methodology, Project administration, Software, Visualization, Writing – original draft, Writing – review & editing. TK: Conceptualization, Investigation, Project administration, Writing – original draft, Writing – review & editing. KT: Investigation, Project administration, Writing – original draft, Writing – review & editing. SM: Investigation, Project administration, Writing – original draft, Writing – review & editing. TT: Funding acquisition, Project administration, Validation, Writing – original draft, Writing – review & editing, Conceptualization. EP: Conceptualization, Funding acquisition, Methodology, Project administration, Resources, Supervision, Validation, Writing – original draft, Writing – review & editing, Data curation.
