# Supplementary material for: Executive and memory dysfunction related to binge drinking in stroke survivors during a 9-year follow-up
Source: Front Aging Neurosci. 2024 Mar 15;16:1360236. doi: 10.3389/fnagi.2024.1360236 (PMC10978674; doi:10.3389/fnagi.2024.1360236)
Supplement: Supplementary file 1 [file Table_1.DOCX]

Supplementary Table I. Main effects of binge drinking and follow-up time on cognitive performance of binge-drinking and non-binge-drinking

stroke patients at three follow-up assessments compared with the repeated-measures ANOVA. Normalized Z-scores at three follow-ups.

|  | Binge-drinking patients, N = 22 | | | Non-binge-drinking patients, N = 63 | | | Binge drinking  F_1,83_ | | Follow-up time F_2,166_ | |
| --- | --- | --- | --- | --- | --- | --- | --- | --- | --- | --- |
|  | 1^st^ follow-up | 2^nd^ follow-up | 3^rd^ follow-up | 1^st^ follow-up | 2^nd^ follow-up | 3^rd^ follow-up | P | η_p_^2^ | P | η_p_^2^ |
| **Executive function** |  |  |  |  |  |  |  |  |  |  |
| Phonemic fluency | -0.439 | -0.710 | -1.220 | 0.482 | 0.063 | -0.289 | **0.002** | 0.113 | **<0.001** | 0.246 |
| Trail Making Test† | -1.773 | -1.798 | -1.585 | -0.399 | -1.299 | -0.541 | **0.013** | 0.073 | **<0.001** | 0.690 |
| Stroop Test† | -0.286 | -0.322 | -0.739 | -0.324 | -0.409 | -0.689 | 0.924 | 0.000 | **0.005** | 0.063 |
| **Memory** |  |  |  |  |  |  |  |  |  |  |
| WMS LM I Immediate | -0.191 | -0.627 | -1.197 | 0.093 | -0.259 | -0.773 | 0.130 | 0.028 | **<0.001** | 0.266 |
| WMS LM II Delayed | -0.184 | -0.585 | -0.936 | 0.153 | -0.098 | -0.654 | 0.190 | 0.021 | **<0.001** | 0.299 |
| 10-word list learning | -1.075 | -1.372 | -1.713 | -0.290 | 0.089 | -0.817 | **0.002** | 0.112 | **<0.001** | 0.133 |

†A square-root transformation was used in the analysis. Trail Making Test = difference score of parts B and A; Stroop Test = difference score of interference and naming parts; WMS = Wechsler Memory Scale; LM I Immediate = Logical Memory I, Immediate Recall; LM II Delayed = Logical Memory II, Delayed Recall). The 1^st^ follow-up took place at 6 months, the 2^nd^ follow-up at 2 years and the 3^rd^ follow-up at 9 years poststroke. η_p_^2^ = Eta-squared, effect size.
